# Properties of Lime-Cement Concrete Containing Various Amounts of Waste Tire Powder under Different Ground Moisture Conditions

**DOI:** 10.3390/polym14030482

**Published:** 2022-01-25

**Authors:** Leili Mohammadifar, Hania Miraki, Aida Rahmani, Soheil Jahandari, Bahareh Mehdizadeh, Haleh Rasekh, Parisa Samadi, Bijan Samali

**Affiliations:** 1 Faculty of Architectural Engineering, Islamic Azad University, Kerman Branch, Kerman 1167, Iran; leilimohammadifar@gmail.com; 2 Department of Civil Engineering, Iran University of Science and Technology, Tehran 6846, Iran; hania_miraki@alumni.iust.ac.ir (H.M.); samadi_parisa@cmps2.iust.ac.ir (P.S.); 3 Centre for Infrastructure Engineering, Western Sydney University, Penrith, NSW 2751, Australia; aida.rahmani63@gmail.com (A.R.); b.samali@westernsydney.edu.au (B.S.); 4 School of Civil and Environmental Engineering, University of Technology Sydney, Sydney, NSW 2007, Australia; bahareh.mehdizadehmiyandehi@student.uts.edu.au

**Keywords:** lime-cement concrete, rubber powder, groundwater, curing times, geotechnical properties

## Abstract

Lime-cement concrete (LCC) is a non-structural concrete in which lime and cement are used as the main binders. However, although LCC has many applications in reducing the settlement of foundations and providing a support layer for shallow foundations, little research has been conducted to evaluate its behaviour in various moisture conditions. Previous researchers have studied the feasibility of using waste tires in conventional concrete to alleviate their negative environmental impacts. However, in field projects, rubber has not been widely used because its application leads to the strength reduction of concrete. In the case of LCC, attaining high strengths is not required and thus application of waste tire particles sounds reasonable. This research evaluated the impact of various rubber powder contents on the fresh, geotechnical and durability properties of LCC at different saturation degrees induced by the capillary action and groundwater level increment, which has not been studied before. The results of more than 320 tests showed that the application of tire powder increases workability and decreases the water absorption of LCC. Moreover, all 60-day cured specimens exposed to 100% saturation degree experienced a strength reduction of less than 10% by using rubber powder contents varying from 0 to 20%. Moreover, increasing the saturation degree from 0 to 100% decreased the average compressive strength by 13.5 and 22% for 60-day cured samples of two different mix designs. The results of this research confirm that LCC containing up to 10% rubber powder could be promisingly used underneath or close to the groundwater table without its strength and geotechnical properties being jeopardized due to rubber employment and/or exposure to ground moisture.

## 1. Introduction

The rapid growth in population and consequently in the vehicle numbers has led to the annual generation of approximately 1.5 billion tons of waste tires [1,2]. The majority of waste tires are landfilled, leading to the occupation of a vast area of land. Part of waste tires are burnt off without being efficiently re-utilized [3], leading to toxic fumes being released. Either burning or storing waste tires can bring about adverse environmental impacts, including soil, water, and air pollution, and eventually pose significant threats to human and animal health. Getting advantage of the waste tires in civil engineering projects can effectively reduce the adverse impacts caused by the accumulation of waste tires [4,5,6,7,8].

The use of scrap tire rubber as high-strain-capacity materials in road construction can improve the impact resistance, fatigue performance, and toughness of the base and subbase of roads [9]. The addition of rubber particles has potential benefits and multiple disadvantages. Although crumb rubber inclusion leads to compressive and tensile strength reduction, structural applications of crumb rubber may still be practicable if appropriate contents of rubber aggregates are involved. Crumb rubber provides low absorption, superb water resistance with low shrinkage, acid resistance, and excellent sound and thermal insulation. Additionally, with the inclusion of tire aggregates, the materials can undergo large deformations without total disintegration and easily absorb substantial plastic energy [2,10]. Mechanical properties, fatigue performance, and damage characteristics of rubber-modified recycled aggregate concrete were studied by Liu et al. [11]. The results showed that the flexural strength, compressive strength, and elasticity modulus of the rubber-modified recycled aggregate concrete decreased; however, the peak strain, peak deflection, and ultimate strain increased by increasing the rubber content such that the ultimate strain of rubber-modified recycled aggregate concrete increased by 3.45 times by the addition of rubber (20% of the sand). Guo et al. [12] also concluded that by increasing the rubber content, the fracture toughness and fracture energy of recycled aggregate concrete reinforced by steel fiber-crumb rubber first increased and then decreased. Li et al. [7] found that, although increasing the rubber content reduced the compressive strength, flexural strength, and the density of recycled-aggregate concrete, it increased the concrete’s dynamic factor and toughness index.

Concrete is considered the most widely used construction material in the world [13,14,15,16,17,18,19,20,21,22]. Contrary to structural concretes in which achieving high strength values is of special importance, in the case of lime-cement concrete (LCC), as non-structural concrete, attaining high strengths is not required. Hence, a strength reduction of up to approximately 10% due to the use of rubber particles is not a critical issue in LCC. In some warm areas, geotechnical engineers broadly utilize lime concrete (LC) and LCC under the shallow foundations of low-rise buildings to provide support layers for superficial foundations and eliminate the tree roots’ growth. Moreover, LC and LCC columns (also known as stone columns) were cast in soft soils to reduce the settlement of foundations in mid-rise structures [23,24,25]. However, a recent comprehensive investigation conducted by the authors on LCC and LC indicated the remarkable merits of LCC over LC when they are used near or below the groundwater table [24]. Furthermore, the potential of LC and LCC as the alternatives for cement concrete for even structural purposes has been reported, but the investigations have not been adequately developed [26]. However, it is important to note that differentiating LC and LCC from lime-stabilized soils, lime-based mortars, and lime-hemp concretes is very important because they are thoroughly different from each other in every aspect, including mix design, sample preparation, and application.

**Figure 1:**
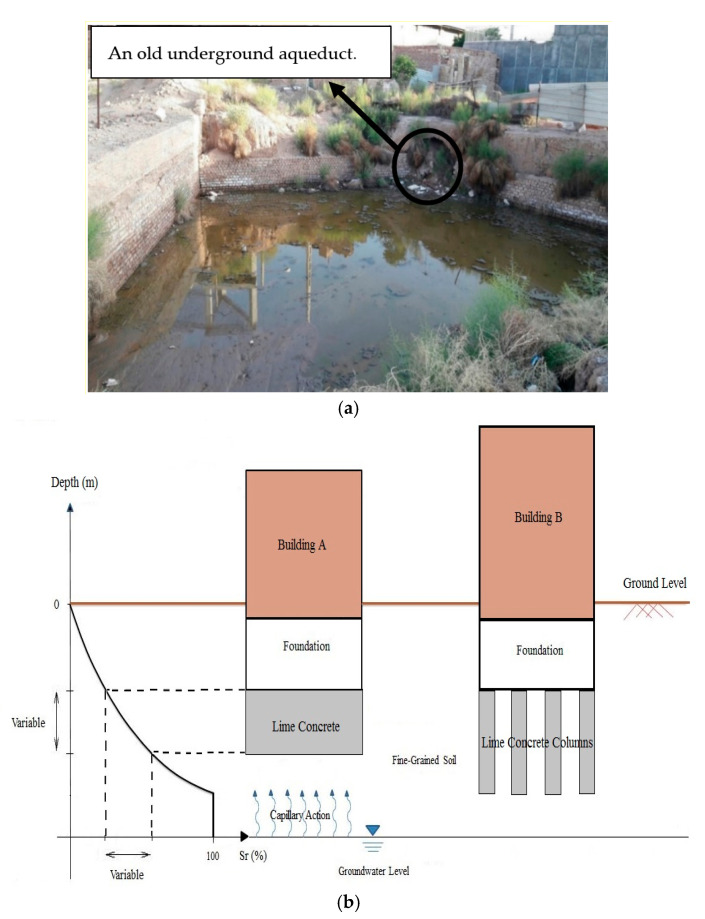
Water penetration into lime-based concrete; (**a**) a dug land exposed to the rise in groundwater level and aqueducts in Kerman city; and (**b**) The effect of the capillary phenomenon on two buildings constructed over LCC and LCC columns [23].

To attain an adequate depth and provide an appropriate base for constructing a foundation, LC (in regions with higher temperature) and LCC could be employed for low-rise buildings up to about three stories (Figure 1a). For mid-rise structures with typically less than seven stories (Figure 1b), LC columns (in areas with higher temperature) and LCC columns are pretty applicable. For the structures with more than seven stories, deep foundations such as piles could be more proper, applicable, and secure [23]. The findings of a previous study indicated that by getting advantage of LCC, the principal settlement in the construction site decreased from 1 m to approximately 20 cm [27]. In a research study on the stiffness and stable deformation of lime-cement-improved soils, the addition of lime and cement led to an enhancement in both strength and resilient modulus and a remarkable decline in the plastic strain [28]. The results of another study revealed that LC columns containing 22% clay and 20% lime brought about a significant increase in the bearing capacity and a considerable decrease in the settlement of weak soil [29].

Low moistures and high temperatures bring about better geotechnical properties for lime-based concrete (i.e., LC and LCC) [30,31,32]. Therefore, lime-based concrete and lime-based concrete columns are broadly utilized in warm weather areas as a support layer beneath the shallow foundations of low- and mid-rise structures. This suggests that getting advantage of LC and LCC in warm regions is reasonable regarding the low precipitation and the long distance between the groundwater table and the foundation’s bottom. Nevertheless, in numerous warm districts worldwide, such as Kerman city in Iran, although the area is located in a warm region and LC and LCC are being widely used, various reasons have resulted in a relatively rapid rise in the groundwater level. Some of which are the devastation of old underground aqueducts, urbanization, destruction of old sewerage frameworks, and bowl shape of the base rock beneath Kerman city [10,11]. From one perspective, the groundwater table in Kerman city is being increased in spite of being located in a warm district. From another standpoint, Kerman’s soil is mainly composed of fine-grained soils, predominantly clayey soil [23,33]. Therefore, on account of the high capillary suction in this type of fine-grained soil, water penetration into lime-based concretes and lime-based concrete columns is quite probable, leading to remarkable strength reduction resulting in adverse impacts on the foundation and, consequently, on the structure. Nonetheless, despite all these reasons, there is still a wrong notion among many engineers about applying LC and LC columns beneath the foundations in Kerman city. Moreover, vast amounts of waste tires are becoming a global threat to the environment. Hence, taking advantage of this waste material can alleviate the concerns and adverse environmental impacts associated with their disposal. However, it needs to be assured whether or not the involvement of the rubber powder jeopardizes the fresh, mechanical and durability properties of LCC, particularly while it is exposed to different ground moisture conditions. All of the abovementioned challenges necessitate the conduction of this research.

A review of previous research studies indicated that even though numerous investigations were performed on the lime-stabilized soils, lime-based mortars, and lime-hemp concrete, few research studies were carried out on the lime-based concretes. In addition, although LCC has many applications in reducing the settlement of foundations in mid-rise buildings and providing a support layer for shallow foundations in low-rise buildings, little research has been conducted to evaluate their behaviour in various ground moisture conditions. This is while in many districts capillary suction or rise in the groundwater table is highly probable to reach LC and LCC, which eventually results in adverse impacts on the foundations and, consequently, on the structures. Additionally, no previous study has been conducted to evaluate the effects of waste tire powder on the fresh, geotechnical, and durability properties of LC and LCC.

Hence, the current study aims to evaluate the effects of various RPCs, curing periods, and degrees of saturation on the workability, water absorption, compressive strength and many other geotechnical properties of two LCC mixes. It is clear that waste tires can be promisingly used in the field if the replacement of natural aggregates with rubber powder does not significantly jeopardize the fresh, mechanical and durability properties of LCC. However, getting advantage of this waste material can alleviate the concerns and adverse environmental impacts associated with waste tires’ disposal, particularly in some regions where vast amounts of waste tires are available.

## 2. Materials

### 2.1. Soils and Aggregates

**Figure 2:**
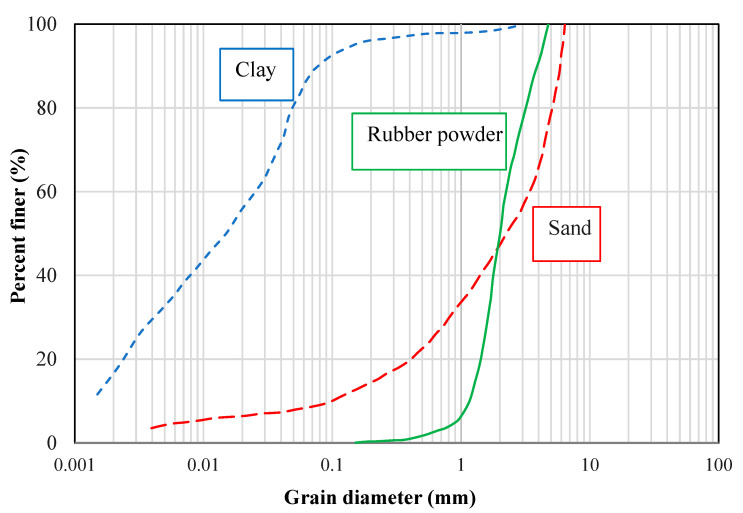
Grain size distribution curves of sand, rubber powder, and clay.

Figure 2 outlines the grain size distribution curves of the used rubber powder, clay (CL), and sand (SW-SM) used in this study. Table 1 indicates the geotechnical properties of the clayey and sandy soils. In the present study, rubber was obtained from Kerman Barez Tire Factory (Kerman, Iran), and the coarse-grained soil was obtained from Ekhtiar Abad Sand Mine of Kerman, Iran, where all the coarse-grained soils used for lime concrete (LC) projects in Kerman city are sourced from. This coarse-grain soil was extensively employed in several other investigations [23,29,34]. Furthermore, digging the construction sites all over Kerman city (Iran), the layers of fine-grained soil (mostly clayey soil) are situated from the land surface to almost 30 m below the ground surface. For the LC and LCC projects in Kerman (Iran), the same type of clayey soil extracted from the construction site is often used. Hence, in order to better represent the clayey soil properties used in the real LCC projects, a building site in Kerman city (Iran) was selected for the clayey soil samples. Accordingly, both the coarse-grained soil and clayey soil utilized in this study are the same as those utilized in real LC and LCC projects in Kerman city, Iran. Prior to specimen preparation for the main tests, the soils were placed in an oven at a temperature of 105 °C until a constant mass was obtained. According to Table 2, the clayey soil is mainly composed of 41.75%, 15.15%, and 5.21% of SiO_2_, Al_2_O_3_, and Fe_2_O_3_, respectively, with a cumulative amount of 62.11%, which is lower than 70% (the minimum amount required for pozzolanic materials) [35]. Hence, enhancing the unconfined compressive strength (UCS) as well as reducing the shrinkage of the concrete or swelling of clayey soil by introducing pozzolanic materials is important [10,36].

### 2.2. Binders

Hydrated lime (also known as Artiman Ahak Type A), with a purity of over 70%, in compliance with the requirements of ASTM C977-18 [44], was purchased from Artiman Ahak Company in Kerman, Iran, and Portland cement (equivalent to ASTM Type I) was obtained from Momtazan Cement Factory of Kerman (Kerman, Iran). Chemical compositions of clay, cement, and lime obtained from X-ray Fluorescence (XRF) analysis are indicated in Table 2. As it can be observed, lime mainly comprises calcium oxide, which is in accordance with the results of previous research [45], and cement predominantly comprises silica and calcium oxide [23]. Lime and cement were passed through a No. 40 sieve prior to be used in the characterization and main tests.

### 2.3. Water

In some previous research studies, it was reported that the water quality can also affect the mechanical properties of concrete and cementitious materials [46,47,48,49,50,51,52,53,54,55]. Therefore, in the current investigation, distilled water was used for the characterization tests. However, tap (drinking) water was utilized for the specimens’ preparation since it is often used in real LCC projects to appropriately represent the actual condition of LCC projects [16,56,57].

## 3. Experimental Program and Methodology

As previously discussed, the objectives of this research study are to investigate the impacts of different rubber powder contents (RPCs) and different moisture conditions induced by the capillary phenomenon and rise in the groundwater table on the geotechnical properties of lime-cement concrete (LCC) with two different mix designs. Hence, the amounts of the materials, sample preparation process, curing condition, and curing time for all of the specimens, as well as all of the conducted tests, including characterizations and UCS tests, were quite the same as those utilized for LCC specimens in the authors’ previous studies [11,21]. However, more details about sample preparation, curing procedure, and compressive strength tests are discussed in the following.

### 3.1. Sample Preparation, Curing, and UCS Test

The optimal content of clayey soil in lime-based concretes is suggested to be in the range of 20–30% of the dry weight of coarse-grained soil based on the National Lime Association and a number of recently published articles [11,12,20,21]. This is due to the fact that lime does not react in mixtures consisting of only coarse-grained soils. Based on the research studies mentioned above, the optimal amount of clay utilized in all mix designs in this research was 23% (of the dry weight of coarse-grained soil). This amount has a significant role in the reaction with lime, provides the chemical bindings among coarse aggregates, and brings about the maximum strength enhancement according to the author’s previous research studies [11,12,20,21].

For the lime-based concrete projects, the amount of binders in field implementations varies from 150 to 200 kg/m^3^ concrete [21]. Regarding the specific weight of the soils utilized in the current research, the total content of binders was kept at 7% of the dry weight of soils (almost 170 kg/m^3^ of concrete) in all samples of the concretes. Accordingly, two groups of specimens were prepared (Group A with 2% cement and 5% lime and Group B with 3% lime and 4% cement). Using a combination of lime and cement instead of using lime alone was based on the results obtained from previous research conducted by the authors [24], which indicated the superb performance of LCC compared with LC in humid conditions. The water content of all mix designs was 24.04% of the total dry weight of all the materials utilized in the mixtures so that the fresh concretes could be appropriately placed into the molds without requiring compaction or vibration. Different percentages of RPCs (0, 5, 10, 15, and 20% of the dry weight of coarse-grained soil) were used as a replacement for coarse-grained soil to prepare the specimens. To prepare the samples, the dry materials were well combined using an automatic stainless steel mixer (Hobart N50-619 Litre 5 Quart Planetary Mixer), supplied by Abadgaran Company, Iran, for approximately three minutes until a homogeneous mixture was achieved. Then, water was added to the mixture, and the blending process was continued for three consecutive minutes until a homogeneous blend was attained.

The fresh concrete was then poured into the cylindrical molds with 70 mm diameter and 140 mm length. After three days of curing, the samples were demolded, followed by measuring their height, diameter, and weight with accuracies of 0.1 mm and 0.01 g, respectively. Subsequently, samples were placed in plastic bags to prevent significant moisture loss during their curing process. The samples were cured in the laboratory environment with a temperature of 20 ± 1 °C and a natural humidity of about 26% for 14, 28, and 60 days. In order to make the results applicable for the field projects, it is better to simulate the real field condition [58,59,60,61,62,63,64,65,66,67,68,69]. In other words, these specific curing times were selected in this research since foundations and structures are often built on lime-based concretes after two to eight weeks of concrete casting [21]. In the last four days of curing, the plastic bags were detached from the samples. The samples were then air-dried for 48 h in the presence of 26% relative humidity followed by being placed in an oven with a temperature of 50 °C for 48 h to dry and to minimize the effect of temperature on the strength development of the samples [30,32,70]. Then, the UCS test was conducted on three dried specimens (for each curing time) to evaluate the compressive strength of the concrete samples which were cured in dry condition, representing the concrete cast in a dry land with a far distance from the groundwater table (Saturation degree (Sr) = 0%). To determine the compressive strength of the specimens at saturated condition (when the groundwater reached the concrete (Sr = 100%)), three samples for each concrete group were submerged in water for 48 h. The specimens were then taken out of the water, surface-dried with a towel, and were subjected to the UCS test. The dry samples were placed in plastic bags to apply partial saturation (when moisture reached the concretes on account of the capillary suction (Sr = 50%)). Then, they received the predetermined moisture content to reach the saturation degrees as mentioned earlier [10,11]. After 48 h that the moisture was fully distributed through the samples and every part of the samples received a constant moisture content, the UCS test was conducted on specimens. To minimize the error in UCS results, three identical samples for each test were prepared and tested, the average of which was reported as the representative value. For the compressive strength tests, a tri-axial test apparatus with a maximum capacity of 28 kN and resolution of 0.026 kN (without any confinement pressure) was employed. The loading rate was set at 1 mm/min as stated by ASTM D 5102-09 [70]. Additional details about sample preparation, curing conditions, the process of specimen saturation, and UCS tests are presented in previous studies carried out by the authors [23,30,31]. The experimental program is presented in Table 3. In total, 320 samples were prepared to test and identify the strength and geotechnical properties of LCC samples at various curing times, saturation degrees and rubber powder contents.

### 3.2. Workability

Workability tests are conducted to determine the fresh properties of concrete. The slump test was primarily designed and used to measure the workability of conventional concrete, and hence it may not be appropriate for other types of concretes such as lime-based concretes. However, it was reported that the flow table test could be a more practical test to evaluate the workability of lime-based mortars and concretes [34,71]. Thus, in the present study, the workability of concrete mixes was measured using a flow table test based on ASTM C270-07 [72]. In general, there is no specified target value for the workability of the samples measured through the flow table test. However, in this research, the amount of water used in the mix designs was determined through the flow table tests in a way that the mixture could be placed inside the molds without the need for vibration or compaction. This reasonable workability was achieved when the diameter of the mixes reached 220 mm on the flow table after the test was performed.

### 3.3. Water Absorption

To study the water absorption of the samples, following ASTM C642 [73], the specimens were initially placed in an oven with a temperature of 105 ± 5 °C for 24 h, followed by measuring their mass (Wd). Afterward, the samples were submerged in water for 48 h, and once again, their mass was measured (Ws). Eventually, the percentage of water absorption was calculated as follows:

(1) $$\mathrm{Water}\;\mathrm{absorption}\;\left(\%\right)=\frac{\mathrm{Ws}-\mathrm{Wd}}{\mathrm{Wd}}\times 100.\;$$

### 3.4. Geotechnical Properties

The geotechnical properties of tested specimens are presented in Table 4 and Table 5. The investigated parameters in this section were considered important parameters while designing lime-based concretes. It is also worth mentioning that the utilized equations and factors are all applicable to either lime concrete (LC) or lime-cement concrete (LCC) based on the previous research studies [23,24,30,31,32,34,74,75].

For unreinforced concretes where the material exhibits a non-linear behaviour or where the initial tangent modulus does not provide sufficient needed information, it is preferred to investigate secant modulus (E_s_). In other words, a secant modulus is used to study the resistance of lime-based concretes to deformation, which is measured by dividing 50% of the UCS to the corresponding axial strain [76].

One of the crucial considerations for structural and non-structural concretes is ductility, which describes the capability of the concretes to have deformations and certain energy dissipation to avert abrupt brittle failure when they are subjected to extreme loads, earthquakes, and winds. Therefore, in this research, the deformability index (I_D_) of the specimens was determined, representing the deformation properties of the specimens which are calculated using Equation (2) [77,78].

(2) $$I_{D}=\varepsilon_{f}/\varepsilon_{\mathrm{cu}}$$

where $\varepsilon_{f}$. is the failure strain of the specimens for any duration of curing at each saturation degree with any RPC content of 0, 5, 10, 15 and 20%, and $\varepsilon_{\mathrm{cu}}$. outlines the failure strain corresponding to each specimen at the same curing time and saturation degree with 0% RPC.

Bulk modulus defines the elastic deformation of the concrete when it undergoes pressure on all axes; in other words, it measures the resistance of the samples to compression and is calculated through the following equation [79]:

(3) $$K=\sigma /(\Delta V/V)=\sigma /(\varepsilon_{\mathrm{xx}}+\varepsilon_{\mathrm{yy}}+\varepsilon_{\mathrm{zz}})=E_{s}/3(1-2\vartheta )$$

where (ΔV/V) signifies the volumetric change, σ is hydrostatic pressure, $\varepsilon_{\mathrm{xx}}$, $\varepsilon_{\mathrm{yy}}$ and $\varepsilon_{\mathrm{zz}}$ stand for the direct strains parallel to the x, y, and z axes respectively, ϑ outlines the Poisson’s ratio, which is taken as 0.3 in this research, and $E_{s}$ outlines the secant modulus. Bulk modulus is usually used to predict the elastic properties, the shrinkage of the soil at the initial ages, the cracking caused by plastic shrinkage, and plastic settlement [80].

In order to investigate the elastic response of soil and concrete to stress and repeated loads, resilient modulus should be evaluated. It needs to be mentioned that numerous parameters, including soil type (for instance, coarse or fine-grained), loading condition, moisture content, density, etc., have considerable impacts on the resilient modulus [81,82]. Thompson (1966) suggested calculating the resilient modulus using Equation (4) [83]:

(4) $$M_{R}\;\left(\mathrm{MPa}\right)=0.124\times \mathrm{UCS}\;\left(\mathrm{kPa}\right)+68.8.\;$$

In order to investigate the resistance of specimens to transverse deformation shear modulus of the samples was calculated. Since shear modulus is of particular importance for structural design and analysing the site response, disregarding this parameter may lead to severe damage and loss [84]. Shear modulus can be calculated through the following equation [85]:

(5) $$G\;(\mathrm{MPa})=\frac{\sigma_{\mathrm{xy}}}{\varepsilon_{\mathrm{xy}}+\varepsilon_{\mathrm{yz}}}=\frac{\sigma_{\mathrm{xy}}}{2\varepsilon_{\mathrm{xy}}}=\frac{\sigma_{\mathrm{xy}}}{\gamma_{\mathrm{xy}}}=E_{s}\frac{\mathrm{MPa}}{2\;\left(1+\vartheta \right)}.\;$$

where${\;\sigma }_{\mathrm{xy}}$, ε, $E_{s}$ and ϑ are shear stress, shear strain, secant modulus, and the Poisson’s ratio, respectively, and $\gamma_{\mathrm{xy}}$ is equal to $\varepsilon_{\mathrm{xy}}$ + $\varepsilon_{\mathrm{yx}}$ [86].

## 4. Results and Discussion

In this section, the effects of rubber powder content (RPC), curing time, two mix designs, and saturation degrees induced by the rise in the groundwater table were evaluated on the properties of lime-cement concrete (LCC) including its workability, water absorption, compressive strength, and geotechnical properties.

### 4.1. Workability

**Figure 3:**
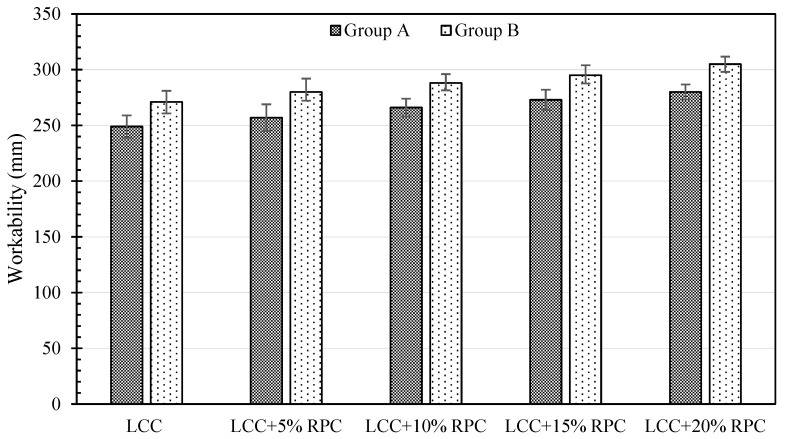
Workability of two groups of concretes containing various amounts of RPC.

From the results of flow table tests presented in Figure 3, it can be observed that the workability of concrete Group A of the samples is lower than that of Group B. The higher workability of Group B samples compared to Group A can be attributed to the fact that the specific gravity of the lime is lower than that of cement, and the surface area of cement is lower than that of lime. Moreover, as observed in Figure 3, the workability of the samples decreases with the increase in RPC, which could be attributed to the hydrophobic characteristics of rubber powder and their substitution with natural aggregates with hydrophilic nature. In other words, the amount of free water was slightly increased in the matrix, which improved the workability. However, from the workability test results, it could be concluded that the workability of all the samples was relatively high enabling the concretes to be placed in the molds without the need for vibration or compaction, which is suitable for practical applications.

### 4.2. Water Absorption

**Figure 4:**
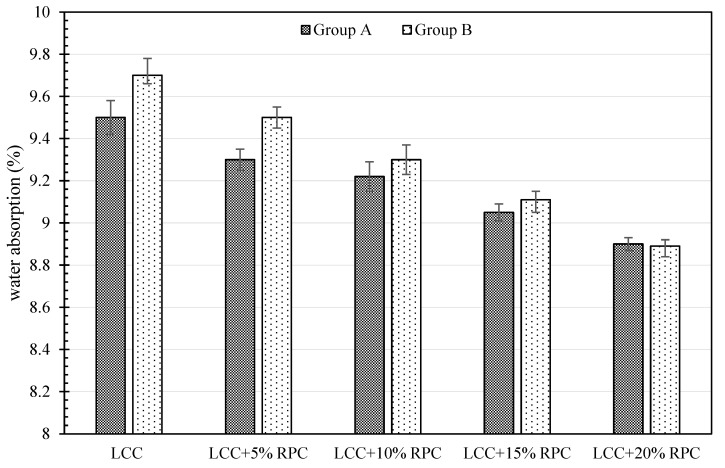
Water absorption rate of two groups of concretes containing various amounts of RPC.

The water absorption rate is often considered a good indication of the durability of concrete and cementitious materials. As observed in Figure 4, Group A and Group B samples had approximately identical water absorption rates. More precisely, water absorption rates of Group A samples were slightly lower compared with Group B. This could be attributed to the higher content of lime in these samples. In other words, the lower specific gravity of lime compared with cement results in the incorporation of the higher volume of lime in the samples. This eventually leads to minor pores in comparison with Group B of the samples, which brings about lower water absorption. In addition, a reduction in water absorption in both groups could be vividly observed, with the increase in RPC. This is also due to the hydrophobic characteristics of rubber powder and their replacement with natural aggregates with hydrophilic characteristics. Moreover, the difference between the water absorption rates of Groups A and B samples decreases with increasing RPC such that in the samples containing 20% rubber powder the water absorption of Groups A and B are approximately equal. This may be because with the increase in RPC some existing pores in Group B samples (due to lower lime content in Group B compared with Group A) become filled, which decreases the difference between the water absorption rates of Groups A and B samples.

### 4.3. Compressive Strength

**Figure 5:**
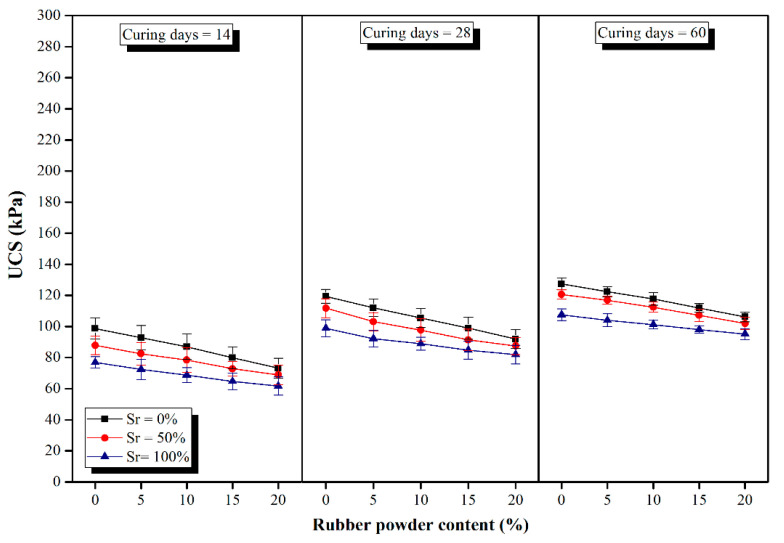
UCS values of Group A samples versus various rubber powder contents at different curing days and saturation degrees.

**Figure 6:**
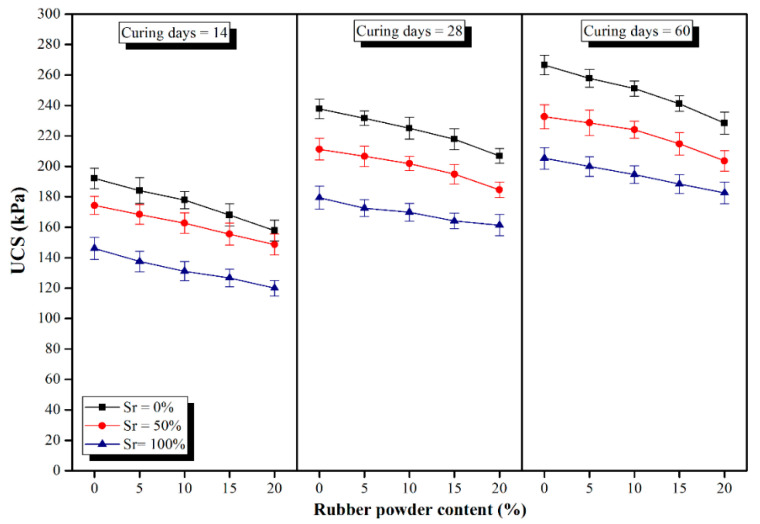
UCS values of Group B samples versus various rubber powder contents at different curing days and saturation degrees.

The UCS test results versus different RPC contents (0, 5, 10, 15 and 20%) at 14, 28 and 60 curing days at different degrees of saturation (0, 50 and 100%) for samples of Group A (2% cement + 5% lime) and Group B (4% cement + 3% lime) are demonstrated in Figure 5 and Figure 6, respectively. Based on the UCS test results, RPC does not have a positive impact on UCS such that the higher the RPC, the lower the UCS. Nevertheless, since lime-based concretes are not used as structural concretes, they are not required to gain high strength values. Generally, the UCS values of lime-based concretes are much lower than those of ordinary Portland cement (OPC) concretes. The lower strength values of lime-based concrete compared to OPC concrete could be attributed to: first, much lower binder contents used in lime-based concretes (150–200 kg/m^3^); second, the presence of large content of clay and soil, and third the water/binder ratio is much higher in lime-based concretes compared with OPC concrete to achieve required workability. It should be noted that the low amounts of binders in lime-based concretes often provide the required strength suggesting that using a higher amount of binder to gain higher strength values is not required nor economical. In addition, the brittleness index of concrete mostly increases with strength increase, which gives rise to crack propagation in case of settlement of sub-soil under the foundations.

In a previous study on the effects of saturation degrees and durations of curing on the compressive strength of lime concrete (LC), it was concluded that the mentioned parameters have considerable effects on the compressive strength of the samples [23,30]. Additionally, the outcome of a similar study demonstrated that the inclusion of moisture led to significant influences on the bearing capacity of LC columns cast in soft clayey soils [29]. Nevertheless, the findings of a recent research study by Jahandari et al. [24] revealed that by replacing 3% of lime in lime concrete (LC) with cement (replacing LC with LCC) the impacts of moisture conditions on the UCS values of the samples decreased significantly while the duration of curing was still an influential factor. Hence, in this research, LCC with two distinct mix designs was considered to be studied. The comparison between the results of Group A with Group B samples indicated that with similar RPCs, curing days, and degrees of saturation, the UCS values of Group A are nearly two times those of Group B. Thus, within the constant amount of binder, by increasing the lime content and decreasing the cement content, the UCS of LCC samples decreases. A similar result was also reported by Lollini et al. [86].

As previously discussed, higher RPCs, led to lower strength values. At a constant degree of saturation, the UCS reduction rate at different curing periods and various amounts of rubber powder was nearly constant. The UCS values of Group A samples at saturation degree of 0% and curing time of 14 days decreased by 5.96, 11.89, 18.98 and 25.84% by increasing the RPC from 0 to 5, 10, 15 and 20%, respectively. Similarly, for the same samples cured for 28 days, the percentages of reduction in compressive strength were 6.27, 11.70, 17.15, and 23.02%, respectively in comparison to the control sample. The same trend was observed for samples in Group B. Li et al. [87] also reported a reduction in the compressive strength, flexural strength, and density of recycled aggregate concrete by increasing the rubber content. The strength reduction by the employment of higher amounts of rubber is due to the following reasons:

(1) Rubber powder has lower strength values compared with its matrix around. Hence, when the concrete is subjected to external forces, the cracks first appear in the contact zone of the concrete matrix, and then they gradually initiate propagation until the concrete crumbles. Such discrepancy in the performance makes rubber particles act like voids in concrete [88,89,90,91,92,93,94,95].
(2) Rubber particles have a remarkably low modulus of elasticity compared with natural aggregates [90,96,97,98].
(3) The hydrophobic nature of the rubber particles brings about imperfect adhesion and bonding between the cement paste and rubber particles which leads to further weakening of the mechanical performance of the entire concrete [4,91,99,100].

The results demonstrate that although increasing the RPC in LCC led to compressive strength reduction, LCC containing up to 10% rubber powder could be promisingly used underneath or close to the groundwater table without its strength and geotechnical properties being significantly jeopardized as a result of rubber employment and/or exposure to moisture. Using RPC up to 10% reduced the strength value by approximately 10% in many specimens. Moreover, all 60-day specimens exposed to 100% saturation degree had a strength reduction of less than 10% by using multiple contents of rubber powder varying from 5 to 20%. Additionally, strength reduction by using higher RPCs was comparatively lower in Group B of the samples than Group A, implying that using higher cement content and lower lime content mitigates the negative impact of rubber in LCC specimens.

The compressive strength of specimens increased with increasing the curing period due to the hydration reactions. Nevertheless, insignificant increases in strength values were obtained after a curing period of 28 days, which was in line with the findings of previous studies [23,24]. In other words, all of the samples gained more than 80% of their 60-day strength at a curing period of 28 days, for instance, the 28-day cured sample of Group B with 10% RPC at 50% Sr had a strength value of 201.86 kPa, which was 90% of its 60-day UCS value.

As shown in Figure 5 and Figure 6, the UCS values at different curing times and various rubber contents decreased by increasing the degree of saturation which could be attributed to the partial disintegration of the chemical bonds between lime and clayey soil. Although strength reduction due to the saturation increment was higher for samples in Group B compared with Group A, the ultimate UCS values of Group B samples, even in the condition of full saturation, were greatly higher than those obtained for samples of Group A, implying their superb resistance in the presence of moisture. To illustrate, by increasing the Sr from 0 to 50%, 8.50% reduction in strength (from 177.87 to 162.75 kPa) was observed for the 14-day cured samples of Group B with 10% RPC; also, further increasing the Sr from 50 to 100%, brought about a linear decrease of 19.42% in the compressive strength of samples (from 162.75 to 131.13 kPa). While in the case of Group A samples, the reductions were respectively 9.76% (from 86.96 to 78.44 kPa) and 12.42% (from 78.44 to 68.69 kPa). As observed, although strength reduction due to the increase in saturation degree is comparatively higher for samples of Group B, their ultimate strength after full saturation is more than twice those from Group A. Moreover, the strength reduction due to the RPC increment is lower at higher degrees of saturation implying the superb resistance of rubber powder in the presence of moisture. For instance, at 0% Sr, the 14-day cured samples of Group A experienced a strength reduction of 25.84% by increasing the RPC from 0 to 20%, while at 100% Sr the reduction was 16.69%. Similar trends were obtained for the other specimens.

### 4.4. Stress–Strain Behaviour and Geotechnical Properties

**Figure 7:**
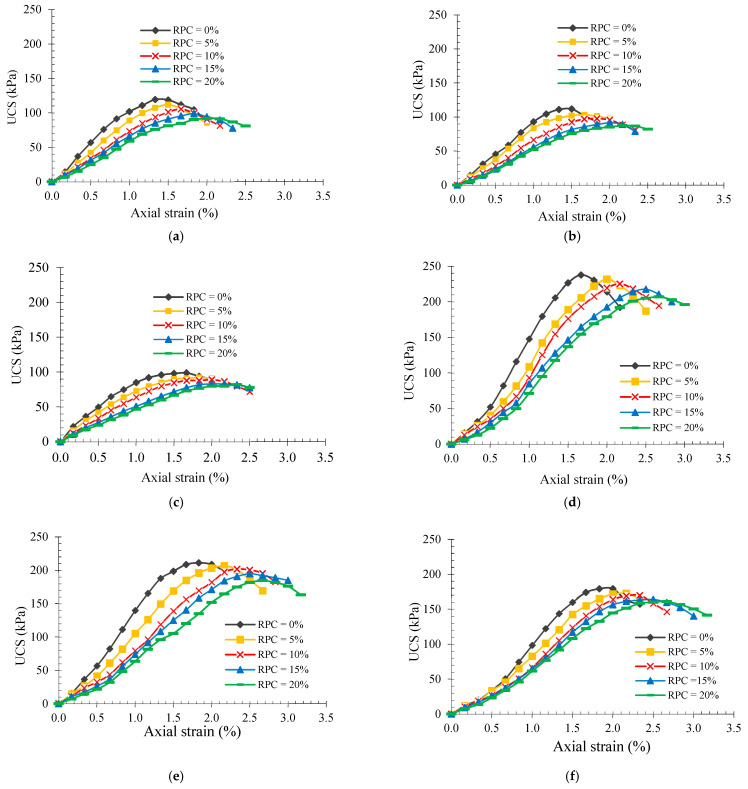
Stress–strain curves of 28-day cured samples (**a**) Sr = 0%, (**b**) Sr = 50% and (**c**) Sr = 100%, Group A; (**d**) Sr = 0%, (**e**) Sr = 50% and (**f**) Sr = 100%, Group B.

The stress–strain curves of 28 day-cured of the specimens from Groups A and B at different moisture conditions are demonstrated in Figure 7. The stress–strain curves of the 14-day and 60-day cured tested specimens are not illustrated in this study as similar trends were observed for all the specimens cured at various ages. Nonetheless, all the geotechnical properties of the specimens tested in this research were calculated and outlined in Table 4 and Table 5 for Group A and Group B of the specimens, respectively.

Comparing the stress–strain curves of Groups A and B of the specimens shows that the failure strains for Group A of the specimens are lower than those of Group B specimens. This could be due to the fact that lime has a lower specific weight in comparison to cement. As mentioned before, the total percentage of binder in this study was a constant value of 7%, so that the volume of the binder when only 2% cement combined with 5% lime was used was higher than the binder volume when 4% cement combined with 3% lime was employed. Moreover, since the water content in all mix designs was a constant value of 24.04%, Group A experienced lower workability compared with Group B, which led to lower failure strains.

Based on the results presented in Table 4 and Table 5, extending curing days and lowering degrees of saturation (Sr) resulted in higher secant modulus for all of the samples. Such behaviour is attributed to the moisture reduction and progress of pozzolanic reactions by extending the curing duration. For instance, at saturation degree of 50% and RPC of 10%, extending the duration of the curing period from 14 to 60 days for samples in Groups A and B increased the secant modulus from 3.37 to 6.74 MPa and from 6.51 to 12.25 MPa, respectively. In addition, lowering the saturation degree from 100 to 50%, resulted in an increase in the secant modulus from 4.39 to 5.10 MPa and from 7.83 to 9.40 MPa respectively for the 60-days cured samples of Groups A and B including 20% RPC. Moreover, increasing RPC led to lower secant modulus, for instance, at 0% saturation degree and 28 day-curing periods, increasing RPC from 0 to 20% led to a reduction in the elastic modulus of samples in Groups A and B from 11.48 to 5.82 MPa and from 14.15 to 8.27 MPa, respectively. This is due to the lower elasticity modulus of rubber, as previously discussed.

As shown in Figure 7, the failure strains of all of the samples at all curing periods increased slightly when the saturation degree increased and/or the curing period decreased. As an example, for 28-day cured samples with 10% RPC, an increase in the saturation degree from 0 to 100% resulted in an increase in the failure strains from 1.67 to 2.00% and 2.17 to 2.33%, for Groups A and B of the samples, respectively. Similar trends were also observed in other studies [23,101]. Obtained trends confirm the more ductility of the samples at higher moisture contents. Additionally, the use of rubber in the LCC led to higher strain values. For instance, at 0% degree of saturation, the strain values of 28-day cured samples of Group A increased by 12.78, 25.56, 37.59, and 50.37% when RPC content increased from 0 to 5, 10, 15, and 20%, respectively. The strain increment helps the concrete absorb more energy by undergoing deformation when external loads are applied, which mitigates the propagation of internal cracks and consequently prevents cracks from spreading across the entire volume.

As observed in Table 4 and Table 5, for a given curing time, increasing the saturation degree brought about higher failure strains and subsequently higher deformability indexes for the majority of samples. Besides that, at a given saturation degree, by extending the RPC, the deformability index of samples decreased, the reasons of which were previously discussed. To illustrate, at the saturation degree of 100%, by increasing the RPC from 5 to 20%, the deformability index of 60-day cured samples from Group A increased from 1.11 to 1.44. Nevertheless, no specific trend for deformability index was perceived by increasing the saturation degree and curing time.

The samples could resist the swelling and shrinkage of clayey soil in the mixtures because of cement contribution and its coexistence with lime. Such behaviour could be attributed to the filling of large volumes of the pores of sandy soil by the clayey soil along with the pozzolanic activities of lime and the cementation reactions from strong inter-particle bonding. Similar behaviour was observed in previous research studies where the introduction of cement to LC brought about a remarkable impact on the susceptibility of the LC to saturation and let the soil preserve its strength in the presence of moisture [10,23,102].

A direct relationship between secant modulus and bulk modulus is apparent from Equation (4). Hence, extending the duration of curing, decreasing RPC content, and lowering the saturation degree resulted in an increase in the bulk modulus of all the studied concretes. As shown in Table 6, after 60 days of curing, when the saturation degree increased from 0 to 100%, the bulk modulus of Groups A and B of the samples with 10% RPC decreased from 6.54 to 4.61 MPa and from 12.55 to 8.11 MPa, respectively. A similar decreasing trend was observed for bulk modulus by decreasing the duration of curing. At 0% saturation degree, by reducing the curing time from 60 to 14 days, the bulk modulus of Groups A and B of the specimens with 5% RPC decreased from 7.67 to 3.87 MPa, and from 14.32 to 7.10 MPa, respectively. Moreover, for 28-day cured samples of Groups A and B at 0% saturation degree, increasing the RPC content from 0 to 20% reduced the bulk modulus from 9.57 to 4.85 MPa and from 11.79 to 6.89 MPa, respectively.

The correlation between resilient modulus and UCS values of the samples is linear and direct as confirmed with Equation (5). Thus, extending the duration of curing, increasing the RPC content, and lowering the saturation degree resulted in an increase in the resilient modulus of all the studied concretes. As presented in Table 6, after 60 days of curing, when the saturation degree increased from 0 to 100%, the resilient modulus of Groups A and B of the specimens with 10% RPC decreased from 83.39 to 81.35 MPa, and from 99.93 to 92.93 MPa, respectively. Furthermore, at Sr = 0%, by extending the curing time from 14 to 60 days, resilient modulus of Groups A and B of the samples with RPC of 0% increased from 81.03 to 84.60 MPa, and from 92.63 to 101.85 MPa, respectively. Additionally, similar to bulk modulus and secant modulus, increasing the curing time and RPC, as well as decreasing the saturation degree leads to an increase in the shear modulus of all the tested specimens.

## 5. Conclusions

Lime-cement concrete (LCC), as a non-structural concrete, has been broadly utilized in some warm regions to provide a support layer beneath the shallow foundations of low-rise buildings. However, fairly limited research has been conducted to evaluate the geotechnical and durability properties of LCC under different ground moisture conditions induced by the capillary action and rise in the groundwater table. On the other hand, several research studies were conducted on getting the advantage of waste tire rubber in conventional concretes. However, in field projects, rubber has not been widely used because its employment leads to strength reduction in concrete specimens. For structural concretes, achieving high strength values is of particular importance, but in the case of LCC, attaining high strengths is not required. Hence, in this research, the impact of various rubber powder contents (RPC), saturation degrees, curing times and two mix designs on the workability, geotechnical and durability properties of LCC were studied. The following main conclusions can be drawn:

(1) Within a constant amount of binder, by increasing the lime content and decreasing the cement content, the UCS value decreased such that the UCS values of Group B samples were nearly two times those of Group A. Increasing the degree of saturation brought about a reduction in the UCS values at different curing times and various rubber powder contents. This could be attributed to the partial disintegration of the chemical bonds between lime and clayey soil. However, strength reduction due to the saturation increment was higher for samples from Group B compared with Group A; nevertheless, the ultimate UCS values of Group B samples, even in the condition of full saturation, were greatly higher than those obtained for the samples of Group A, implying their superb resistance in the presence of moisture.
(2) LCC prepared with the mix design of Group B samples containing up to 10% rubber powder could be promisingly used in dry conditions and even underneath or close to the groundwater table with insignificant impact on the compressive strength and geotechnical properties because of rubber employment and/or exposure to moisture. Therefore, in some cities where vast amounts of waste tires are available, taking advantage of this waste material can not only provide reasonable resistance for LCC but can also alleviate the concerns and adverse environmental impacts associated with their disposal.
(3) However, it should be noted that LCC samples with the mix designs of Group A are considered suitable to be cast in a totally dry land where there is a long distance between the bottom of the concrete and groundwater level or where the moisture from any direction cannot reach the concrete due to the capillary suction, etc. It is reported that the capillary action can take place and moisturize the clayey soils even up to 25 m above the groundwater level. Therefore, in the construction sites where the type of soil is predominantly clayey soil (such as Kerman city in Iran), the use of LCC prepared with the mix designs of Group A is not considered rational. Nevertheless, it is obvious that the capillary suction is considerably limited in coarse-grained soils so that they could be used where the type of soil beneath the foundation is a coarse-grained soil with high drainage capacity.
(4) The performance of LCC (containing rubber powder) against multiple wet–dry and freeze–thaw cycles has not been studied in this study; thus, it is recommended for future research to investigate the durability of LCC containing rubber powder against wet–dry and freeze–thaw cycles. Moreover, the results of ongoing research conducted by the authors indicated the superb performance of fly ash and wood ash when they are used as cement or lime replacements in lime-based concretes exposed to different ground moisture conditions. Thus, it is also recommended to investigate the use of these by-products in such lime-based concretes containing rubber powder.

## Figures and Tables

**Table 1:** Properties of soils [24].

Characteristics	Results	Used References
Coarse-grained soil type (sand)	SW-SM	[37]
Effective size (D_10_)	0.10	[38]
Uniformity coefficient (C_u_)	34	[38]
Coefficient of curvature (C_c_)	1.88	[38]
Plastic limit (PL)	22%	[39]
Liquid limit (LL)	27%	[40]
Plasticity index (PI)	5%	[41]
Fine-grained soil type (clay)	CL	[37]
Mineral of CL	Kaolinite	[41]
Activity degree (A) of CL	0.47	[41]
D_10_ of CL	0.0015	[38]
C_u_ of CL	18	[38]
C_c_ of CL	0.40	[38]
PL of CL	23%	[39]
LL of CL	34%	[40]
PI of CL	11%	[41]
Optimum water content (ω_opt_) of CL	15%	[42]
Maximum dry density (γ_d_) of CL	18.75 kN/m^3^	[42]
Specific gravity (G_s_) of CL	2.47	[43]

**Table 2:** Oxide compositions of the materials.

Component Oxides	Composition of Clay (%)	Composition of Lime (%)	Composition of Cement (%)
CaO	13.20	73.70	63.41
SiO_2_	41.75	1.15	21.63
Al_2_O_3_	15.15	0.11	4.21
Fe_2_O_3_	5.21	0.27	3.12
MgO	5.15	1.60	2.81
SO_3_	3.48	0.02	2.61
NaCl	0.08	0.01	-
Loss on ignition	12.58	23.15	0.81

**Table 3:** Experimental program.

Variables and Constants	Values
Cement (%) + Lime (%)	2 + 5; 4 + 3
Rubber powder content (by dry weight of the coarse-grained soil (%))	0, 5, 10, 15, 20
Saturation degrees (%)	0, 50, 100
Curing times (days)	14, 28, 60
Clay (by the total dry weight of the coarse-grained soil and rubber powder (%))	23
Water (by total dry weight of all the materials (%))	24.04

**Table 4:** Geotechnical properties of Group A of the specimens.

Curing Period (Day)	Sr (%)	RPC (%)	UCS (kPa)	ε_f_ (%)	E_s_ (MPa)	I_D_	K (MPa)	M_R_ (MPa)	G (MPa)
14	0	0	98.66	1.83	5.39	-	4.49	81.03	2.07
	0	5	92.78	2.00	4.64	1.09	3.87	80.30	1.78
	0	10	86.93	2.16	4.02	1.18	3.35	79.58	1.55
	0	15	79.93	2.33	3.43	1.27	2.86	78.71	1.32
	0	20	73.17	2.50	2.93	1.37	2.44	77.87	1.13
	50	0	87.91	2.00	4.40	-	3.66	79.70	1.69
	50	5	82.44	2.16	3.82	1.08	3.18	79.02	1.47
	50	10	78.44	2.33	3.37	1.17	2.81	78.53	1.29
	50	15	72.82	2.50	2.91	1.25	2.43	77.83	1.12
	50	20	68.93	2.67	2.58	1.34	2.15	77.35	0.99
	100	0	76.82	2.17	3.54	-	2.95	78.33	1.36
	100	5	72.32	2.33	3.10	1.08	2.59	77.77	1.19
	100	10	68.69	2.50	2.75	1.15	2.29	77.32	1.06
	100	15	64.67	2.67	2.42	1.23	2.02	76.82	0.93
	100	20	61.69	2.83	2.18	1.31	1.82	76.45	0.84
28	0	0	119.41	1.33	11.48	-	9.57	83.61	4.42
	0	5	111.92	1.50	9.17	1.13	7.64	82.68	3.53
	0	10	105.44	1.67	6.76	1.25	5.63	81.87	2.60
	0	15	98.93	1.83	6.60	1.38	5.50	81.07	2.54
	0	20	91.92	2.00	5.82	1.50	4.85	80.20	2.24
	50	0	111.77	1.50	9.25	-	7.70	82.66	3.56
	50	5	103.06	1.67	7.98	1.11	6.65	81.58	3.07
	50	10	97.67	1.83	6.42	1.22	5.35	80.91	2.47
	50	15	91.42	2.00	5.52	1.33	4.60	80.14	2.12
	50	20	87.44	2.17	5.14	1.44	4.28	79.64	1.98
	100	0	98.82	1.66	9.88	-	8.24	81.05	3.80
	100	5	92.18	1.83	8.38	1.10	6.98	80.23	3.22
	100	10	88.94	2.00	6.66	1.20	5.55	79.83	2.56
	100	15	82.82	2.17	5.17	1.31	4.31	79.32	1.99
	100	20	81.93	2.33	4.88	1.41	4.06	78.96	1.88
60	0	0	127.41	1.16	10.98	-	9.15	84.60	4.22
	0	5	122.40	1.33	9.20	1.15	7.67	83.98	3.54
	0	10	117.66	1.50	7.84	1.29	6.54	83.39	3.02
	0	15	111.91	1.67	6.71	1.44	5.59	82.68	2.58
	0	20	106.15	1.83	5.80	1.58	4.83	81.96	2.23
	50	0	120.54	1.33	9.06	-	7.55	83.75	3.49
	50	5	116.89	1.50	7.79	1.13	6.49	83.29	3.00
	50	10	112.41	1.67	6.74	1.25	5.62	82.74	2.59
	50	15	107.16	1.83	5.86	1.38	4.88	82.09	2.25
	50	20	101.91	2.00	5.10	1.50	4.25	81.44	1.96
	100	0	107.55	1.50	7.17	-	5.98	82.14	2.76
	100	5	103.92	1.67	6.23	1.11	5.19	81.69	2.40
	100	10	101.17	1.83	5.53	1.22	4.61	81.35	2.13
	100	15	97.92	2.00	4.90	1.33	4.08	80.94	1.88
	100	20	95.17	2.17	4.39	1.44	3.66	80.60	1.69

**Table 5:** Geotechnical properties of Group B of the specimens.

Curing Period (Day)	Sr (%)	RPC (%)	UCS (kPa)	ε_f_ (%)	E_s_ (MPa)	I_D_	K (MPa)	M_R_ (MPa)	G (MPa)
14	0	0	192.15	2.00	9.61	-	8.01	92.63	3.70
	0	5	184.14	2.16	8.53	1.08	7.10	91.63	3.28
	0	10	177.87	2.33	7.63	1.17	6.36	90.86	2.94
	0	15	168.10	2.50	6.72	1.25	5.60	89.64	2.59
	0	20	157.78	2.67	5.91	1.34	4.92	88.36	2.27
	50	0	174.37	2.16	8.07	-	6.73	90.42	3.10
	50	5	168.41	2.33	7.23	1.08	6.02	89.68	2.78
	50	10	162.75	2.50	6.51	1.16	5.43	88.98	2.50
	50	15	155.51	2.67	5.82	1.24	4.85	88.08	2.24
	50	20	148.63	2.83	5.25	1.31	4.38	87.23	2.02
	100	0	146.14	2.33	6.27	-	5.23	86.92	2.41
	100	5	137.51	2.50	5.50	1.07	4.58	85.85	2.12
	100	10	131.13	2.67	4.91	1.15	4.09	85.06	1.89
	100	15	126.64	2.83	4.47	1.21	3.73	84.50	1.72
	100	20	119.88	3.00	4.00	1.29	3.33	83.67	1.54
28	0	0	237.74	1.67	14.15	-	11.79	98.28	5.44
	0	5	231.62	2.00	11.24	1.20	9.37	97.52	4.32
	0	10	225.10	2.17	10.14	1.30	8.45	96.71	3.90
	0	15	217.83	2.50	9.23	1.50	7.69	95.81	3.55
	0	20	206.84	2.67	8.27	1.60	6.89	94.45	3.18
	50	0	211.23	1.83	13.20	-	11.00	94.99	5.08
	50	5	206.61	2.17	10.33	1.18	8.61	94.42	3.97
	50	10	201.86	2.33	8.41	1.27	7.01	93.83	3.23
	50	15	194.87	2.50	7.92	1.36	6.60	92.96	3.05
	50	20	184.63	2.67	7.05	1.45	5.87	91.69	2.71
	100	0	179.36	2.00	9.75	-	8.12	91.04	3.75
	100	5	172.60	2.16	8.38	1.08	6.98	90.20	3.22
	100	10	169.88	2.33	7.52	1.17	6.26	89.87	2.89
	100	15	164.00	2.50	7.01	1.25	5.85	89.15	2.70
	100	20	161.00	2.67	6.78	1.34	5.65	88.81	2.61
60	0	0	266.50	1.33	20.04	-	16.70	101.85	7.71
	0	5	257.81	1.50	17.19	1.13	14.32	100.77	6.61
	0	10	251.06	1.67	15.06	1.25	12.55	99.93	5.79
	0	15	241.17	1.83	13.18	1.38	10.98	98.71	5.07
	0	20	228.34	2.00	11.42	1.50	9.51	97.11	4.39
	50	0	232.58	1.50	15.51	-	12.92	97.64	5.96
	50	5	228.59	1.67	13.69	1.11	11.41	97.15	5.26
	50	10	224.09	1.83	12.25	1.22	10.20	96.59	4.71
	50	15	214.84	2.00	10.74	1.33	8.95	95.44	4.13
	50	20	203.61	2.17	9.40	1.44	7.83	94.05	3.61
	100	0	205.24	1.67	12.29	-	10.24	94.25	4.73
	100	5	199.90	1.83	10.92	1.10	9.10	93.59	4.20
	100	10	194.63	2.00	9.73	1.20	8.11	92.93	3.74
	100	15	188.35	2.17	8.69	1.30	7.24	92.16	3.34
	100	20	182.62	2.33	7.83	1.40	6.52	91.44	3.01

**Table 6:** Compressive strength reduction rate of LCC samples at various curing times and saturation degrees with increasing RPCs contents.

Concrete Type	Curing Times (Day)	Saturation Degrees (%)	Reduction in the Compressive Strength (%)			
			RPC Content (0 to 5%)	RPC Content (0 to 10%)	RPC Content (0 to 15%)	RPC Content (0 to 20%)
Group A	14	0	5.96	11.89	18.98	25.84
	14	50	6.27	11.70	17.15	23.02
	14	100	3.93	7.65	12.17	16.69
	28	0	6.22	10.77	17.17	21.59
	28	50	7.79	12.62	18.21	21.77
	28	100	3.03	6.74	11.10	15.46
	60	0	5.86	10.58	15.82	19.70
	60	50	6.72	10.00	14.17	17.09
	60	100	3.38	5.93	8.95	11.51
Group B	14	0	4.17	7.43	12.52	17.89
	14	50	2.57	5.32	8.37	13.00
	14	100	3.26	5.79	9.50	14.32
	28	0	3.42	6.66	10.82	14.76
	28	50	2.19	4.44	7.75	12.59
	28	100	1.72	3.65	7.63	12.46
	60	0	5.91	10.27	13.34	17.97
	60	50	3.27	5.29	8.49	10.02
	60	100	2.60	5.17	8.23	11.02

## Data Availability

Data are contained within the article.
